# Genome sequences of *Arthrobacter globiformis* B-2979 phages MidnightRain and Gusanita

**DOI:** 10.1128/MRA.00936-23

**Published:** 2023-11-22

**Authors:** Devansh Bansal, Alexander Z. Fazilat, Matthew V. Genchev, Sanguk Han, David Y. Ho, Amisha Kumar, Brian J. Lam, Noah Q. Le, Ryan P. Lin, Amanda J. Lopez, Nicole Y. Lu, Marco Marroquin, Zainab H. Neemuchwala, William J. Seng, Priya T. Shah, Jason M. Toliao, Christa T. Bancroft

**Affiliations:** 1 Department of Biological Sciences, University of Southern California, Los Angeles, California, USA; 2 Department of Quantitative and Computational Biology, University of Southern California, Los Angeles, California, USA; Department of Biology, Queens College, Queens, New York, USA

**Keywords:** bacteriophage genetics, bacteriophages, bioinformatics, bacteriophage lysis, genomics, integrase, tRNA, immunity repressor

## Abstract

Phages MidnightRain and Gusanita, with siphovirus morphology, were isolated on *Arthrobacter globiformis* B-2979. MidnightRain’s genome consists of 53,674 bp, encoding 101 putative genes and 1 tRNA, whereas Gusanita’s genome is 42,742 bp, encoding 68 putative genes and 2 tRNAs.

## ANNOUNCEMENT

**Fig 1:**
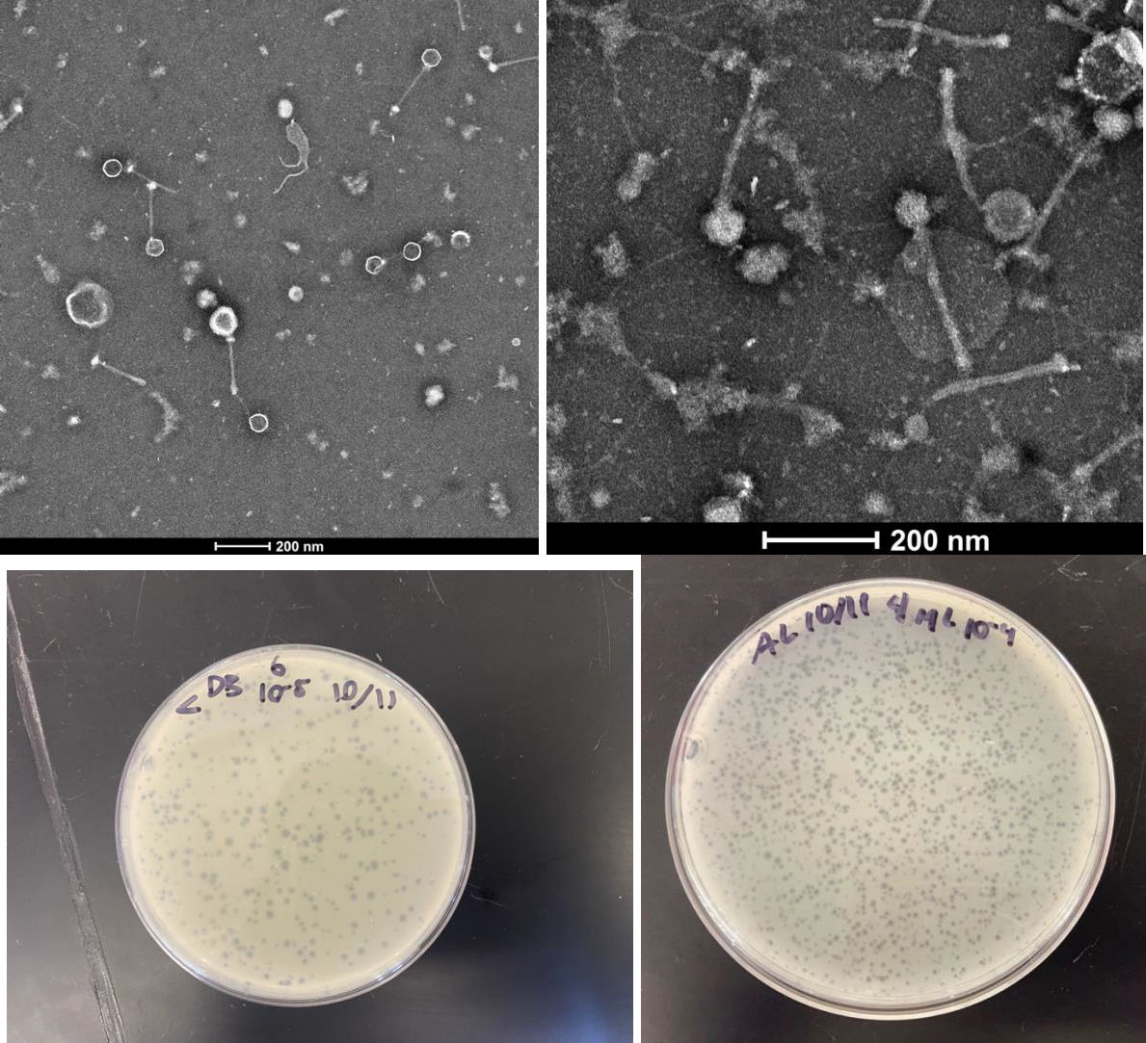
Nano-W negative stain (2) transmission electron micrographs (Talos F200CG2, 200 keV) and plaque for MidnightRain (top left and bottom left) and Gusanita (top right and bottom right). Scale bar is 200 nM.

Thought to be the most abundant biological entity on Earth, bacteriophages, or phages, are viruses that infect and replicate within bacterial hosts (1). Isolating and examining bacteriophages allows us to gain a greater understanding of their biology and elucidate their interactions with their host. Phages MidnightRain and Gusanita were isolated from damp soil samples 1 inch below the ground surface at the University of Southern California in Los Angeles, CA (34.01717 N, 118.28575 W and 34.01897 N, 118.28519 W, respectively) using standard procedures (https://seaphagesphagediscoveryguide.helpdocsonline.com/home). Briefly, each soil sample was washed in peptone-yeast extract-calcium medium, the wash filtered (0.22 µm), then inoculated with *Arthrobacter globiformis* B-2979 and incubated with shaking at 30°C for 48 h. The culture was refiltered, diluted, and plated in soft agar containing *Arthrobacter globiformis* B-2979. After 24 h at 30°C, both MidnightRain and Gusanita produced small clear plaques, 1–2 mm in diameter for MidnightRain and 0.5–1.0 for Gusanita (Fig. 1). Both phages were purified with three rounds of plating before being imaged by negative-stain transmission electron microscopy. Both phages have siphovirus morphology with MidnightRain possessing a 200–202 nm tail and capsid diameter of 54–55.5 nm (*n* = 3), while Gusanita has a 260–265 nm tail and a capsid diameter of 66–67.5 nm (*n* = 3) (Fig. 1).

Double-stranded DNA was purified from high-titer phage lysate using the Promega Wizard DNA cleanup kit, prepared for sequencing using the NEB Ultra II kit, and sequenced on an Illumina MiSeq (v3 reagents). Sequencing reads, detailed in Table 1, were assembled using Newbler v2.9 (3) and checked for accuracy and genomic termini using Consed v29 (4), yielding a 53,674 bp genome for MidnightRain and 42,742 bp for Gusanita, both with 3′ single-stranded overhangs.

**TABLE 1:** Sequencing data and genome characteristics for MidnightRain and Gusanita

Phage	MidnightRain	Gusanita
Number of sequencing reads	264,115	210,771
Length of sequencing reads	150-base single-end	150-base single-end
Coverage of sequencing reads	699×	702×
Genome length	53,674 bp	42,742 bp
GC%	62.5	64.9
Genome end types	3′ single-stranded overhangs (5′-CGCCGGTGA-3′)	3′ single-stranded overhangs (5′-TCCGCCGCGTGA-3′)
Cluster assignment	AY	FF

The genome sequences were auto-annotated using DNAMaster v5.23.6 (cobamide2.bio.pitt.edu) embedded with GeneMark v2.0 (5) and Glimmer v3.02 (6). Following auto-annotation, Starterator (http://phages.wustl.edu/starterator/http://phages.wustl.edu/starterator/) was used to refine suggested start sites. MidnightRain and Gusanita encode 101 and 68 open reading frames, respectively, and 1 and 2 tRNAs, respectively, identified by Aragorn v1.2.38 (7) and tRNAscan-SE (8). Default parameters were used for all software. Based on gene-content similarity of at least 35% to phages in the actinobacteriophage database (9), MidnightRain and Gusanita are assigned to phage clusters AY and FF. HHPred [databases: PDB_mmCIF70_18_Jun, Pfam-A_v36, UniProt-SwissProt-viral70_3_Nov_2021, and NCBI_Conserved_Domains(CD)_v3.19] (10), NCBI BLASTp nr database (11), and Phamerator database Actino_Draft (12) were used to deduce the products of predicted genes. Both phages encode a putative immunity repressor gene, a putative tyrosine integrase (MidnightRain potentially encodes two), and putative excise and are therefore likely temperate phages despite both forming clear plaques similar to other cluster AY and FF phages (9). The genomes of both phages are similarly organized, with predicted structure and assembly genes occupying the right arm of each genome while predicted DNA metabolism genes occupy the left arm. Most of the genes are transcribed rightward except for a few genes in the center of each genome, including the putative tyrosine integrase, that are transcribed leftward.

## Data Availability

MidnightRain is available at GenBank accession OR475247, SRA experimental read SRX20165770, and Illumina read SRR24377452. Gusanita is available at GenBank accession OR195044, SRA experimental read SRX20165764, and Illumina read SRR24377458.
